# Practice report on a credit-based internship for Medical Sciences students: Examining educational effects through fundamental skills for working adults and qualitative content analysis

**DOI:** 10.20407/fmj.2026-006

**Published:** 2026-09-25

**Authors:** Akiko Watanabe, Manabu Totsuka

**Affiliations:** Department of Career Education, Fujita Health University, School of Medical Sciences, Toyoake, Aichi, Japan

**Keywords:** Internship, Medical Sciences students, Generic skills, Career development, Professional identity

## Abstract

**Objectives:**

This study examined the educational outcomes of a credit-based internship program for third-year university students majoring in Medical Sciences, focusing on changes in their Fundamental Skills for Working Adults and career awareness.

**Methods:**

Participants were 57 third-year students from the Faculty of Medical Sciences at University A enrolled in a credit-based internship course. On completion of the internship, a web-based questionnaire was administered. The survey included self-evaluations of Fundamental Skills for Working Adults, Likert scales to evaluate subjective learning outcomes, and open-ended responses. Quantitative data were analyzed using descriptive statistics, and qualitative data were categorized using qualitative content analysis.

**Results:**

Forty-two students responded to the questionnaire (response rate: 73.7%). For Fundamental Skills for Working Adults, more than 80% reported improvements in Proactivity, Active Listening, Situational Awareness, and Discipline. Subjective evaluations showed high overall satisfaction, with 97.6% of respondents reporting being either “very satisfied” or “satisfied,” and 95.2% reporting changes in future vision and career awareness. Qualitative analysis yielded five categories, including “multifaceted understanding of medical settings and workplaces” and “concretization of career visions.”

**Conclusion:**

Credit-based internships can effectively contribute to the development of Fundamental Skills for Working Adults and enhance career awareness among Medical Sciences students.

## Introduction

Early clinical exposure plays a crucial role in shaping the professional identity of university students majoring in Medical Sciences and enhancing their learning motivation.^1,2^ However, Japanese medical universities typically follow a structure in which basic subjects are taught in the early years and specialized subjects, along with practical training, in the later years, limiting opportunities for students to proactively consider their careers. In the latter half of their studies, Japanese university students enter a transitional structure where they undertake mass job-hunting for a limited time. Thus, third-year students often lack sufficient time to assess their developing professional aptitude and career vision. Furthermore, this transition from university to workplace is associated with challenges in career formation and increased anxiety.^3^

Internships in Japan have historically been oriented toward corporate recruitment and selection, with limited emphasis on educational objectives.^4^ Within this context, credit-based internships embedded in university curricula are considered valuable opportunities for students to concretize their aptitudes and career vision.

Advances in information technology (IT) and artificial intelligence (AI), along with demographic changes, have significantly altered the demands placed on medical and technical professions.^5^ Reforms in physicians’ working styles have promoted task shifting and sharing, increasing the need for interdisciplinary collaborative skills, autonomous decision-making, and the need for all medical professionals to adapt rapidly to cutting-edge technologies.^6,7^

This study, therefore, aimed to examine the impact of a credit-based internship program for third-year students in the Faculty of Medical Sciences at University A on career awareness and self-perceived Fundamental Skills for Working Adults.

## Participants and Methods

### Overview of the internship course

The internship course comprised 90 hours (two credits; elective subjects) and consisted of pre-internship exercises, on-site work experience (five days), post-internship exercises, and a report session. Work placement sites were selected from facilities and job types that did not overlap with university-based practical training, and included testing centers and medical device companies.

### Study participants

The participants were 57 third-year students from the Faculty of Medical Sciences (training programs for medical technologists, clinical engineers, and radiological technologists) at University A in 2025 who were enrolled in the internship course.

### Survey methods and analysis

A web-based questionnaire survey was administered to all enrolled students on completion of the internship.

### Students’ perceptions of subjective educational effects and learning outcomes

To examine the educational effects of the internship course, six items were assessed: (1) participation motivation, (2) consistency with pre-internship expectations, (3) understanding of jobs and workplaces, (4) degree of goal setting and action plan implementation, (5) changes in future vision and career awareness, and (6) overall course satisfaction. The questionnaire items were developed by the researchers, and responses were obtained using a four- to five-point Likert scale for each item.

The analysis calculated the number and percentage of responses for each option, and trends in subjective educational effects were descriptively analyzed on course completion. As this study was a survey aimed at evaluating educational practices, no statistical hypothesis testing was performed, and the results were organized using descriptive statistics.

### Quantitative evaluation of changes in fundamental skills for working adults

Self-evaluations were obtained using a three-point scale by comparing pre- and post-internship levels for the 12 competency elements of the “Fundamental Skills for Working Adults for the 100-Year Life Era,”^8^ defined by the Ministry of Economy, Trade, and Industry. For each competency element, responses were categorized as “improved,” “no change,” or “declined,” and the number and percentage of responses per option were calculated. Descriptive statistics were used to identify trends in changes for each element.

### Qualitative analysis of learning content

Open-ended responses were sought regarding achievement of internship participation goals and reasons, knowledge and skills acquired from the internship, and newly recognized personal strengths derived from participating in the internship. A qualitative content analysis was performed by interpreting the meaning of the descriptions and categorizing them based on similarities. To ensure objectivity, the categories were finalized after discussions among multiple faculty members.

### Ethical considerations

This study was reviewed and approved by the Institutional Review Board of Fujita Health University and was approved by the university president (HM25-659, approval date: March 23, 2026).

Participants were verbally informed about the study purpose, the potential use of the collected data in reports and presentations at academic conferences, their right to refuse participation, the voluntary nature of responses, and the independence of the survey from course evaluation. This information was also provided in writing at the beginning of the questionnaire. Consent was obtained through questionnaire submission. Participants were informed that no personal information would be collected and that submitted data could not be deleted.

## Results

Forty-two participants who responded to the questionnaire and consented to participate in the study were included in the analysis (response rate: 73.7%). An overview of the internship participants and questionnaire respondents is presented in Table 1.

High positive response rates were observed for consistency with pre-internship expectations and understanding of jobs/workplaces, both of which reached 100.0% (Table 2). The positive response rate for achievement of participation motivation was 88.1%, whereas that for goal setting and action plan implementation was 61.9%. Changes in future vision and career awareness were reported by 95.2% of respondents, and overall course satisfaction was 97.6%.

### Changes in Fundamental Skills for Working Adults

The results for the 12 competency elements of the Fundamental Skills for Working Adults are shown in Table 3. More than 80% of students reported “improved” for four competencies: Proactivity, Active Listening, Situational Awareness, and Discipline. In contrast, relatively less improvement was observed in Planning, Creativity, Stress Management, and Communication.

### Qualitative analysis results of learning content

Qualitative analysis of the open-ended responses yielded five major categories (Table 4). The most frequently cited categories were descriptions related to “multifaceted understanding of medical settings and workplaces” and “understanding of roles within the medical system and concretization of career visions.” Students reinterpreted the role of technicians beyond merely providing inspection skills. They realized their roles encompass broader professionalism, including patient interaction and interdisciplinary collaboration. Experiences such as mobile diagnostics and corporate operations helped them grasp the overall picture of healthcare. Additionally, exposure to non-hospital settings challenged the assumption that technician careers are limited to hospital employment, serving as an opportunity to concretize their future work aspirations.

## Discussion

### Relationship between educational/learning effects and fundamental skills for working adults

The results revealed extremely high levels of overall satisfaction with the internship course and a high degree of achievement in participation motivation. These outcomes are likely attributable, at least in part, to a high sense of self-efficacy. However, the positive response rate for “goal setting and action plan implementation” was lower than that for other subjective learning outcomes, suggesting the need for additional educational support to help students translate their goals into concrete action plans.

This high sense of fulfillment may be related to improvements in Proactivity within Fundamental Skills for Working Adults. Previous studies^9,10^ have shown that career education interventions enhance career decision-making self-efficacy (CDSE) and promote goal setting and execution behaviors. In the present study, the experience of engaging in workplace-based learning and working toward on-site practical goals can be interpreted as supporting the perceived improvements in Proactivity. Furthermore, the affirmative sense of accomplishing planned actions in medical settings may reinforce professional confidence and further promote Proactivity, given self-efficacy has been shown to enhance vocational potential through increased achievement motivation.^11^

### Trends in fundamental skills for working adults

More than 80% of students reported improvement in four competencies: Proactivity, Active Listening, Situational Awareness, and Discipline. This aligns with the qualitative category of “Acquisition of interpersonal skills and professionalism,” reflecting learning gained through on-site patient interaction practices. In contrast, improvements in Planning and Creativity were relatively low. Shinmura^12^ reported a similar decline in competencies related to the “thinking deeply” domain, including problem identification, planning ability, and creativity in project-based internships, a pattern consistent with this study. The prominent improvements in interpersonal and action skills, such as Proactivity and Active Listening, suggest that work-experience internships are effective in forming the foundation for workplace adaptability and collaboration skills as medical professionals.

### Transformation of professional identity and experiential learning processes

The qualitative findings of “multifaceted understanding of medical settings and workplaces” and “understanding of roles of each facility in the medical system and concretization of career visions” substantiate effective functioning through Kolb’s^13^ experiential learning model. Students engaged in “concrete experiences” at off-campus facilities and, through reflective observations, understood how knowledge acquired at the university is applied in real-world settings. This reflective process appears to have altered students’ preconceived notions that hospitals are the primary workplace for technicians and engineers, leading to a more balanced conceptualization of professional roles from a broader medical system perspective. The fact that 95.2% of students reported changes in future vision and career awareness suggests that experiences at non-hospital facilities functioned as boundary-crossing learning. Their experiences provided an opportunity to form a universal identity as medical professionals independent of specific employment settings.

### Limitations of short-term programs and improvement strategies

Jackson^14^ pointed out the difficulty of acquiring higher-order skills such as self-designing and managing processes (Planning) through short-term work experiences. This observation aligns with the limited improvements in Planning and Creativity in this study. Therefore, short-term internships may benefit from supplementary educational components, such as post-internship workshops that integrate practical experiences into medium- to long-term learning plans. Such an approach may also enhance Planning and Creativity.

### Limitations and future challenges

This study has several limitations. First, it relied on self-reported questionnaires administered only post-internship, which may have introduced subjective bias into the results. Second, the response rate was 73.7% (42/57), leaving the motivations and characteristics of non-respondents unclear, potentially resulting in selection bias. Third, the number of respondents varied across training programs, and some groups included only a small number of participants. Therefore, caution is required when generalizing the findings across all Medical Sciences students.

Future research could incorporate pre–post comparative longitudinal designs and combine post-internship workshops with long-term follow-up surveys. These approaches would enable scientific verification of the sustained effects of the internship program and contribute to the establishment of a standard model for medical career education.

## Conclusion

A credit-based internship can promote the formation of professional identity and career awareness through high satisfaction levels and improvements in the Fundamental Skills for Working Adults. Conducting similar longitudinal surveys using the framework of this study and comparing changes over time may provide concrete insights for constructing and improving career education curricula in medical universities.

## Acknowledgment

We express our deepest gratitude to all the host facilities that cooperated in the implementation of this internship program.

## Funding Sources

No financial support was received for this study.

## Conflict of Interest

The authors declare no conflict of interest related to this study.

## Figures and Tables

**Table 1:** Characteristics of internship enrollees and respondents by training program

Training program	Enrollees (n=57)	Respondents (n=42)	Response rate (%)
Medical Technology	31	20 (47.6)	64.5
Clinical Engineering	21	19 (45.2)	90.5
Radiological Technology	5	3 (7.1)	60.0
**Total**	**57**	**42**	**73.7**

**Table 2:** Students’ perceptions of subjective educational effects and learning outcomes

Items	Very positive n (%)	Positive n (%)	Non-positive n (%)	Total n	Positive response rate (%)
Achievement of participation motivation	0 (0.0)	37 (88.1)	5 (11.9)	42	88.1
Consistency with pre-internship expectations	33 (78.6)	9 (21.4)	0 (0.0)	42	100.0
Understanding of jobs/workplaces	35 (83.3)	7 (16.7)	0 (0.0)	42	100.0
Goal setting and action plan implementation	0 (0.0)	26 (61.9)	16 (38.1)	42	61.9
Changes in future vision/career awareness	20 (47.6)	20 (47.6)	2 (4.8)	42	95.2
Overall course satisfaction	33 (78.6)	8 (19.0)	1 (2.4)	42	97.6

Note. Positive response rate was calculated as the combined percentage of “very positive” and “positive” responses. Non-positive responses included all other response options.

**Table 3:** Changes in Fundamental Skills for Working Adults (N=42)

Competency	Sub-competency	Improved n (%)	No change n (%)	Declined n (%)
Action	Proactivity	35 (83.3)	7 (16.7)	0 (0.0)
	Assertiveness	27 (64.3)	15 (35.7)	0 (0.0)
	Execution	33 (78.6)	9 (21.4)	0 (0.0)
Thinking	Problem Identification	25 (59.5)	17 (40.5)	0 (0.0)
	Planning	19 (45.2)	23 (54.8)	0 (0.0)
	Creativity	22 (52.4)	20 (47.6)	0 (0.0)
Teamwork	Communication	23 (54.8)	19 (45.2)	0 (0.0)
	Active Listening	36 (85.7)	6 (14.3)	0 (0.0)
	Flexibility	29 (69.0)	13 (31.0)	0 (0.0)
	Situational Awareness	36 (85.7)	6 (14.3)	0 (0.0)
	Discipline	35 (83.3)	7 (16.7)	0 (0.0)
	Stress Management	22 (52.4)	20 (47.6)	0 (0.0)

**Table 4:** Categorization of learning content and descriptions in internships

Category	Descriptions (Examples)
Multifaceted understanding of medical settings and workplaces	• Learned about overall operation of facilities and workflow from mobile health screenings to secondary examinations.
	• Experienced firsthand differences in atmosphere and roles among hospitals, testing centers, and private companies.
	• Gained a concrete image of my future self by observing professionals in their actual working environments.
Integration of theory and practice	• Engaged in practical experiences not available at the university, such as gastric fluoroscopy, mammography, and identifying abnormal ECG waveforms.
	• Understood how academic knowledge is applied in quality control and multidisciplinary collaboration in clinical settings.
	• Discovered the “realities of medicine” that cannot be learned through classroom study by observing the clinical floor.
Understanding of roles within the medical system and concretization of career visions	• Overcame the stereotype that “technicians only work in hospitals,” broadening career options to include private companies and clinics.
	• Provided a crucial opportunity to establish personal criteria for my future employment.
	• Developed a proactive mindset toward considering my future career path.
Acquisition of interpersonal skills and professionalism	• Realized the importance of pre-examination explanations and communication tailored to each individual patient.
	• Learned that technicians require strong hospitality and collaboration skills in addition to technical testing proficiency.
	• Recognized both the difficulty and the importance of communication skills when interacting with superiors and team members.
Proactive learning motivation and self-transformation	• Became painfully aware of my lack of knowledge in the field, which increased my motivation for self-improvement.
	• Realized the practical necessity of my current studies and felt the need for a study plan focused on national exams and clinical training.
	• Cultivated a proactive learning attitude, such as actively asking questions.
